# Bone Loss around Dental Implants 5 Years after Implantation of Biphasic Calcium Phosphate (HAp/*β*TCP) Granules

**DOI:** 10.1155/2018/4804902

**Published:** 2018-12-05

**Authors:** Vadims Klimecs, Alexanders Grishulonoks, Ilze Salma, Laura Neimane, Janis Locs, Eva Saurina, Andrejs Skagers

**Affiliations:** ^1^Department of Doctoral Studies, Riga Stradins University, Riga, Latvia; ^2^Department of Oral and Maxillofacial Surgery, Riga Stradins University, Riga, Latvia; ^3^Department of Diagnostic Radiology, Riga Stradins University, Riga, Latvia; ^4^Rudolfs Cimdins Centre of Development and Innovations of Biomaterials, Riga Technical University, Riga, Latvia; ^5^Department of Statistic, Riga Stradins University, Riga, Latvia

## Abstract

Biphasic calcium phosphate ceramic granules (0.5–1.0 mm) with a hydroxyapatite and *β*-tricalcium phosphate ratio of 90/10 were used. Biphasic calcium phosphate ceramic granules produced in the Riga Technical University, Riga Rudolph Cimdins Biomaterials Innovation and Development Centre, were used for filling the bone loss on 18 patients with peri-implantitis. After 5 years at the minimum, clinical and 3D cone-beam computed tomography control was done. Clinical situation confirmed good stability of implants without any signs of inflammation around. Radiodensity of the previous gap and alveolar bone horizontally from middle point of dental implants showed similar radiodensity as in normal alveolar bone. This trial is registered with ISRCTN13514478.

## 1. Introduction

Implant-based treatment is a growing part in the modern dentistry. Loss of alveolar bone around dental implants is revealed in 5–10% of patients. A dental implant is considered to be a failure if it is lost, mobile, or shows peri-implant bone loss of greater than 1.0 mm in the first year and greater than 0.2 mm a year after. Peri-implantitis can result in bone loss around the implant and eventual loss of the implant [1]. Peri-implantitis is a site-specific infectious disease that causes an inflammatory process in soft tissues and bone loss around an osseointegrated implant in function [2].

The etiology of the implant infection is conditioned by the status of the tissue surrounding the implant, implant design, degree of roughness, external morphology, and excessive mechanical load.

Regenerative treatment of peri-implantitis also is a growing problem. The optimal result of peri-implantitis treatment is the regeneration of hard and soft tissues supporting the loose dental implant [3–6].

In the peri-implantitis treatment together with operative and conservative treatment, bone substitutes are often used to replace the bone defect; one of the materials is biphasic calcium phosphate (BCP).

BCP is widely used to increase bone after tooth extraction and in dental implantation, also in peri-implantitis treatment.

Calcium phosphates, such as hydroxyapatite (HAp), *β*-tricalcium phosphate (*β*-TCP), and a combination of HAp/*β*-TCP, are used since they do not evoke adverse cellular reactions and, in time, the material is either replaced by bone or integrated into the body, depending on the degradation properties [7–9].

HAp and *β*-TCP or their combination due to their osteoconductivity, crystallographic structures, and chemical composition similar to the skeletal tissue are widely used. They are classified according to their “resorbability,” that is extent of degradation in vivo. HAp has been described as “nonresorbable,” and *β*-TCP has been described as “resorbable.”

BCP bioceramic materials have possibly double action—stable increase of bone in volume and improvement of the remineralization depending on HAp and *β*-TCP relations. HAp strengthens and improves mineralization of the natural bone, which is extremely necessary for good dental implant integration. Radiological densitometric analysis can prove it [3, 10].

The aim of study is to analyze the results of peri-implantitis treatment, where in addition to the classical surgical technique, the bone defect around the dental implant was filled with BCP bioceramic granules. Radiological investigation using 3D cone-beam computed tomography (3D CT) can objectivize the results.

## 2. Materials and Methods

This clinical trial included 18 patients. The main criterion for selecting the patients for this study was the presence of peri-implatitis at any stage, whereas the time of implant placement and the appearance of the first symptoms of peri-implantitis were not taken into account. The age and sex of the patient were not taken into account either.

An important criterion for the selection of the patients was the surgery treatment with addition of BCP, which were developed and produced by Riga Technical University (RTU), Riga Rudolph Cimdins Biomaterials Innovation and Development Centre.

Another important patient selection criterion in this study was the presence of a 3D CT before and after treatment of peri-implantitis. The second, control, 3D CT was done at least 5 years after the treatment.

For the peri-implantitis classification, Riga Institute of Stomatology, Oral and maxillofacial surgery department uses two classifications: Froum and Rosen classification of peri-implantitis [11] and Ata-Ali classification of peri-implantitis [12].

These classifications allow to assess the degree of peri-implantitis, choose treatment tactics, as well as evaluate the quality of treatment.

### 2.1. Peri-Implantitis Treatment

Our patients underwent treatment by the following surgical protocol, which is a reliable and predictable solution in the treatment of progressive peri-implantitis:

- Systemic antibiotics 3 times/day for 2 days before surgery
- Preoperative rinse for 1 minute with a 0.2% chlorhexidine solution
- Local anaesthesia with articaine solution
- Design of mucoperiosteal flap
- Determining the size of the infected area
- Mechanical cleaning, curettage of the implant surface
- The application of a gauze pad moistened with a 2% chlorhexidine solution in the area of bone defect for 5 minutes
- After removing the gauze swab, the defect is washed by 1 g of tetracycline dissolved in 20 ml of sterile physiological solution
- The bone defect filling with bioactive material—HAp/*β*-TCP
- The wound closure with a surgical suture
- Systemic antibiotics 3 times/day for 3 days after surgery

**Figure 1:**
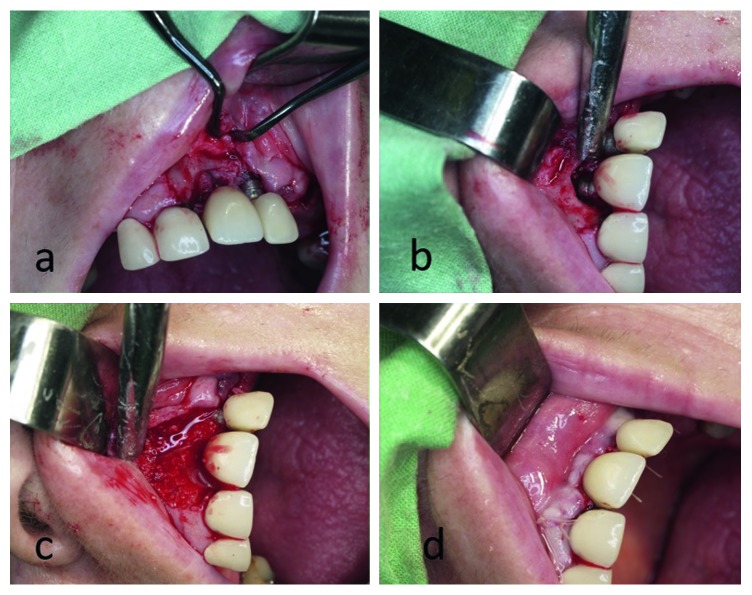
Surgery procedure. (a) Design of mucoperiosteal flap, determining the size of the infected area. (b) Mechanical cleaning, curettage of the implant surface. (c) Insertion of implants composed of HAp/*β*-TCP (ratio of 90/10). (d) Closed wound.

The choice of an antibacterial drug depended on the patient. The first choice of the prescribed drug was Amoxicillin 500 mg; in case of allergies to this drug, Clindamycin 150 mg was chosen (Figure 1).

### 2.2. Source of Implantable Materials

Calcium-deficient hydroxyapatite (CDHAp) was synthesized by an aqueous precipitation technique, where calcium hydroxide and phosphoric acid were used as raw materials in the reaction as follows: Ca(OH)_2_ + H_3_PO_4_/Ca_10_ − *x*(HPO_4_)*x*(PO_4_)_6_ − *x*(OH)_2_ − *x* + H_2_O. The filtered precipitates were formed into granules, dried, and sintered at 1150°C for 2 h. During the sintering process, CDHAp transformed into BCP ceramics with the HAp/*β*-TCP ratio of 90/10. Sintered granules between 0.5 and 1 mm were obtained using vibrational sieves, and the sieved granules were washed in ethanol and dried in a drying oven at 105°C for 24 h. Prior to application, the dried granules were sterilized using steam sterilization.

**Figure 2:**
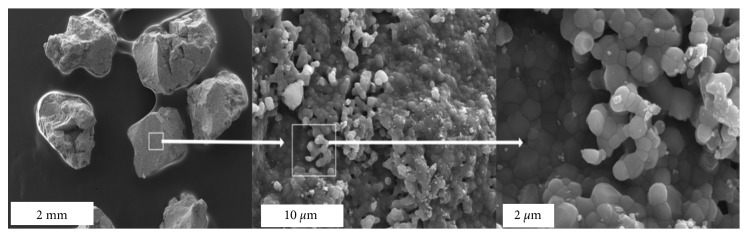
SEM microphotographies of BCP ceramic granules.

SEM images revealed the irregular shape and microstructure of BCP granules. The microstructure is composed of relatively dense structure with grain size *d* < 1 *µ*m and nanosized pores confirming gas adsorption results (Figure 2). Nanopores could be critical for flow of body fluids and protein adsorption [13].

### 2.3. 3D Cone-Beam Computed Tomography

**Figure 3:**
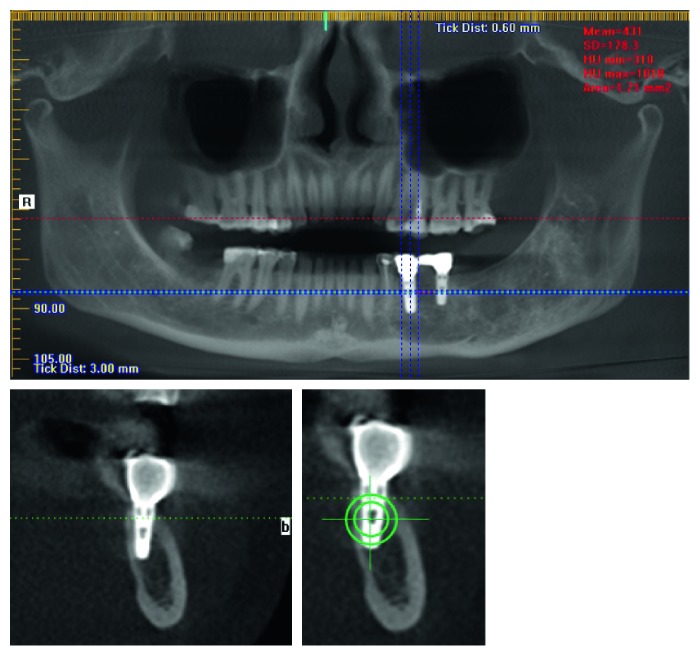
3D CT scans of dental implants. Measuring points for densitometry (green).

The inclusion criterion was the presence of the full series of qualitative 3D CT scans at two time points—one preoperative and one postoperative at least five years after treatment. Modern 3D CT allows obtain images of high quality, while patients are not at risk of high radiation doses [14]. The purpose of second imaging was to monitor the precision and quality of surgery. 3D CT was done with i-CAT Next Generation (KAVO, Germany). Image volume was reconstructed with 0.3 mm voxel size. The tube voltage was 120 kVp, tube current was 5 mA, and exposure time 20 seconds. For image reconstruction, ExamVision program was used (Figure 3).

**Figure 4:**
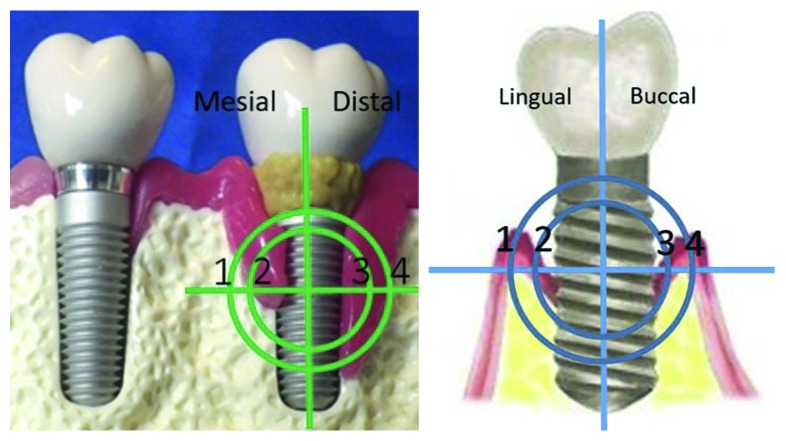
Points of measurement of mineral density (HU).

Densitometry was carried out in 8 points—the first point is the dental implant centre, this is the standard beginning point for all measurements, then 2 points with 2 mm step in mesial and distal direction, as well as 2 points with 2 mm step in buccal and lingual direction. The first point is 2 mm from implant surface, and the second point is 2 mm more from previous measurement point (Figure 4).

## 3. Results

Patients at least 5 years follow-up show good clinical results, which are further confirmed by 3D CT.

To simplify the assessment of the quality of the treatment using the costuming agent based on calcium hydroxyapatite, the percentage of bone tissue loss (the depth of the bone pocket) was taken with respect to the body of the dental implant before and after therapy. In average, bone pocket depth was 34.6% ± 5.4% of the dental implant length and it decreased to 22.3% ± 3.4% after regenerative surgery.

In conformity with Froum classification of peri-implantitis before the treatment, the second stage was revealed in three cases and the third stage in 15 cases. After treatment, the first stage was in 10 cases and the second stage in 8 cases. In conformity of Ata-Ali classification of peri-implantitis the second stage was in one case, the third stage in 3 cases, and the fourth stage in 14 cases, and after treatment, the first stage was in 7 cases and the second stage in 11 cases (Table 1).

Another method to prove the quality of treatment is measurement of bone densitometry. The results of densitometry indicate an improvement in the mineralization of bone tissue quality (Tables 2 and 3).

These parameters are quite stable, as evidenced by Pearson's correlation coefficients.

Pearson's correlation coefficients were calculated between the variables as follows: mediodistally point 1 (mes 1) and mediodistally point 2 (mes 2), mediodistally point 3 (mes 3) and mediodistally point 4 (mes 4), linguobuccally point 1 (ling 1) and linguobuccally point 2 (ling 2), as well as linguobuccally point 3 (ling 3) and linguobuccaly point 4 (ling 4). The following correlations and *p* values were obtained:

  Cor (mes 1, mes 2) = 0.781 (*p* < 0.001)
  Cor (mes 3, mes 4) = 0.443 (*p*=0.065)
  Cor (ling 1, ling 2) = 0.785 (*p* < 0.001)
  Cor (ling 3, ling 4) = 0.582 (*p*=0.011)

**Figure 5:**
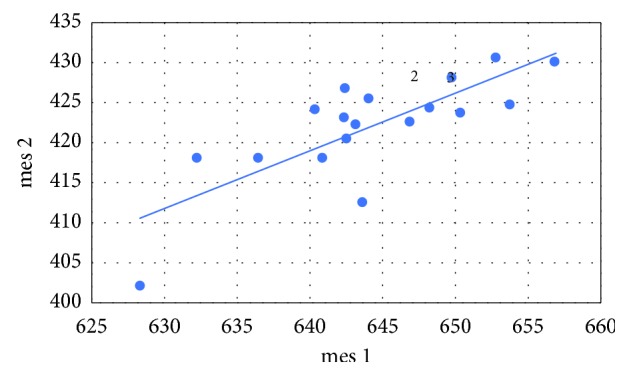
Vertical loss of alveolar bone around dental implants before. Variables mes 1 versus mes 2.

**Figure 6:**
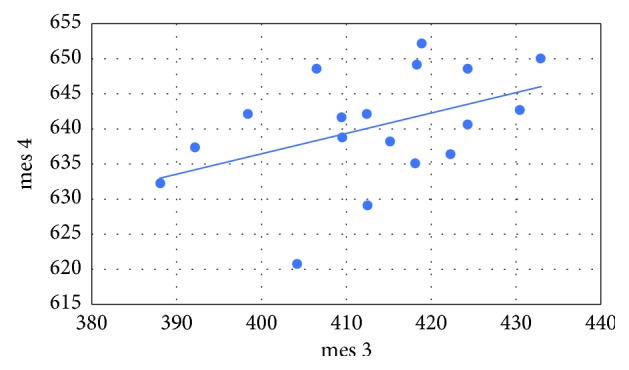
Variables mes 3 versus mes 4.

**Figure 7:**
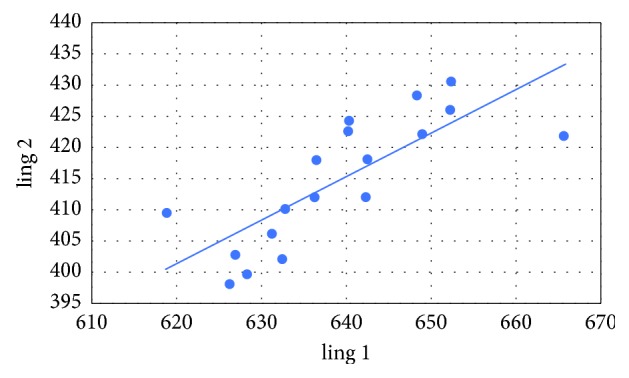
Variables ling 1 versus ling 2.

**Figure 8:**
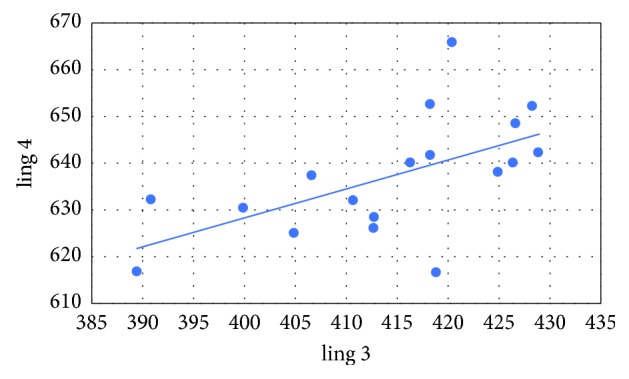
Variables ling 3 versus ling 4.

In all cases, there is a positive relationship (Figures 5–8). At the significance level *α* = 0.05, the Pearson's correlation coefficients between the variables mes 1 and mes 2, ling 1 and ling 2, as well as ling 3 and ling 4 are statistically significant. The only thing that was not statistically significant is Pearson's correlation coefficient between the variables mes 3 and mes 4.

## 4. Discussion

Treatment methods of peri-implantitis are different, and results of investigations and recommendations are contradictory. In general, in the current situation, the surgical therapy with resective and augmentative procedures completes the treatment options. Surgery can be used in order to eliminate peri-implantitis defects, to re-establish hygienic abilities, and to reduce or even stop peri-implantitis progression whereas regenerative therapies may be applicable for defect filling [15, 16]. Not in all studies a benefit for these treatments compared to debridement alone was obtained [17].

We are on the regenerative approach and use BCP materials for replacement of bone defect. The main component HAp was initially considered as bioinert biomaterial till the time when the osteoinductive property was confirmed by Ripamonti [18] and Zhang et al. [19]. Later bioactivity of BCP materials was confirmed [20]. Also regenerative potency of BCP activation of endogenous growth factor TGF*β* after implantation in experiment was obtained [21, 22]. Activation of endogenous osteoprotegerin turns on bone remodelling from osteoclastogenesis to osteoblastogenesis is a positive action of BCP biomaterials in use for recovery of bone defects [23]. Peri-implantitis bone defects, treated in regenerative way in experiments, can be filled by hybrid of living bone and inorganic, but bioactive biomaterial [24]. Our long-term results confirmed effectiveness of synthetic BCP bioceramic materials in regenerative treatment of peri-implantitis.

The unique phase composition and porous structural features of osteoinductive Ca-P ceramics allow to interact with signalling molecules and extracellular matrices in the host system, creating a local environment conductive to new bone formation [25].

The use of 3D CT gives a good, noninvasive approach for determining the depth of peri-implantitis defect and healing of it after therapy. We used 3D CT imaging to confirm the defect after clinical evaluation of the patient and give a glance of it extent.

## 5. Conclusion

Comparing the indices of radiological measurement of the depth of the osseous pockets, radio densitometry of the bone structures before and after treatment of peri-implantitis with the use of pure calcium hydroxyapatite, it may be concluded that long-term results after 5 years are stable. The radiodensity of bone tissue after the application of synthetic biomaterial based on calcium hydroxyapatite differs a little from the intact bone of the patient, which may indicate a high degree of mineralization after implantation of calcium hydroxyapatite crystals.

## Figures and Tables

**Table 1:** Peri-implantitis classification for 18 cases (Froum and Rosen [11] classification and Ata-Ali [12] classification).

Case no.	Dental implant length (mm)	Bone defect before surgery (mm)	Bone defect after surgery (mm)	Froum and Rosen [11] classification before surgery (Stage no.)	Froum and Rosen [11] classification after surgery (Stage no.)	Ata-Ali [12] classification before surgery (Stage no.)	Ata-Ali [12] classification after surgery (Stage no.)
1	10	3	1	2	1	2	1
2	12	8	3	3	2	4	2
3	12	7	2	2	1	4	1
4	10	6	2	3	1	4	1
5	8	5	3	3	1	4	2
6	12	8	4	3	2	3	2
7	10	6	2	3	1	4	1
8	10	6	1	3	1	4	1
9	12	7	2	2	1	4	1
10	14	7	3	3	1	3	2
11	11.5	5	2	3	1	3	1
12	8	6	4	3	2	4	2
13	10	6	3	3	2	4	2
14	9	5	3	3	2	4	2
15	12	7	4	3	2	4	2
16	12	8	5	3	2	4	2
17	10	6	3	3	2	4	2
18	12	7	4	3	2	4	2

**Table 2:** Alveolar bone densitometry (HU) mediodistally (4 points).

Case no.	1st point	2nd point	3rd point	4th point
1	646.8	422.6	388	632.3
2	652.7	430.7	406.4	648.6
3	640.3	424.2	418.1	635.2
4	642.4	426.8	392.2	637.4
5	656.8	430.2	432.8	650.1
6	653.7	424.8	418.3	649.2
7	644	425.6	412.3	642.2
8	650.3	423.8	424.3	648.6
9	649.7	428.2	418.8	652.2
10	648.2	424.4	430.4	642.8
11	642.5	420.6	424.3	640.6
12	636.4	418.1	398.4	642.2
13	640.8	418.1	422.2	636.4
14	642.3	423.2	415.1	638.2
15	628.3	402.1	404.2	620.8
16	643.1	422.3	409.5	638.8
17	643.6	412.6	409.4	641.7
18	632.2	418.2	412.4	629.2

**Table 3:** Alveolar bone densitometry (HU) linguobuccally (4 points).

Case no.	1st point	2nd point	3rd point	4th point
1	665.6	422	420.3	666
2	648.3	428.4	418.2	652.8
3	652.3	430.7	426.3	640.3
4	636.4	418	404.8	625.2
5	648.9	422.2	428.2	652.5
6	642.2	412.2	406.5	637.6
7	631.2	406.2	410.6	632.2
8	642.4	418.2	424.8	638.2
9	652.2	426.1	416.2	640.3
10	640.3	424.4	426.6	648.6
11	636.2	412.2	418.2	641.8
12	626.2	398.2	390.8	632.4
13	632.4	402.2	412.6	626.2
14	640.2	422.8	428.8	642.3
15	632.8	410.3	412.7	628.6
16	628.3	399.8	389.4	616.9
17	626.9	402.8	399.8	630.6
18	618.8	409.6	418.8	616.8

## Data Availability

Data will be available on request, provided that this data does not fall under the directive of the European Union to protect the personal data of patients.
